# A New Perspective on Mechanisms of Neurodegeneration in Experimental Autoimmune Encephalomyelitis and Multiple Sclerosis: the Early and Critical Role of Platelets in Neuro/Axonal Loss

**DOI:** 10.1007/s11481-025-10182-w

**Published:** 2025-02-04

**Authors:** Jacqueline Monique Orian

**Affiliations:** Department of Biochemistry and Chemistry, La Trobe Institute for Molecular Science, School of Agriculture, Biomedicine and Environment, La Trobe University, Bundoora, Vic. 3086 Australia

**Keywords:** Experimental autoimmune encephalomyeltis, Multiple sclerosis, Neurodegeneration, Neuroinflammation, Neuroprotection

## Abstract

Multiple sclerosis (MS) is a central nervous system (CNS) autoimmune disorder, with limited treatment options. This disease is characterized by differential pathophysiology between grey matter (GM) and white matter (WM). The predominant WM hallmark is the perivascular plaque, associated with blood brain barrier (BBB) loss of function, lymphocytic infiltration, microglial reactivity, demyelination and axonal injury and is adequately addressed with immunomodulatory drugs. By contrast, mechanisms underlying GM damage remain obscure, with consequences for neuroprotective strategies. Cortical GM pathology is already significant in early MS and characterized by reduced BBB disruption and lymphocytic infiltration relative to WM, but a highly inflammatory environment, microglial reactivity, demyelination and neuro/axonal loss. There is no satisfactory explanation for the occurrence of neurodegeneration without large-scale inflammatory cell influx in cortical GM. A candidate mechanism suggests that it results from soluble factors originating from meningeal inflammatory cell aggregates, which diffuse into the underlying cortical tissue and trigger microglial activation. However, the recent literature highlights the central role of platelets in inflammation, together with the relationship between coagulation factors, particularly fibrinogen, and tissue damage in MS. Using the experimental autoimmune encephalomyelitis (EAE) model, we identified platelets as drivers of neuroinflammation and platelet-neuron associations from the pre-symptomatic stage. We propose that fibrinogen leakage across the BBB is a signal for platelet infiltration and that platelets represent a major and early participant in neurodegeneration. This concept is compatible with the new appreciation of platelets as immune cells and of neuronal damage driven by inflammatory cells sequestered in the meninges.

**Graphical Abstract:**

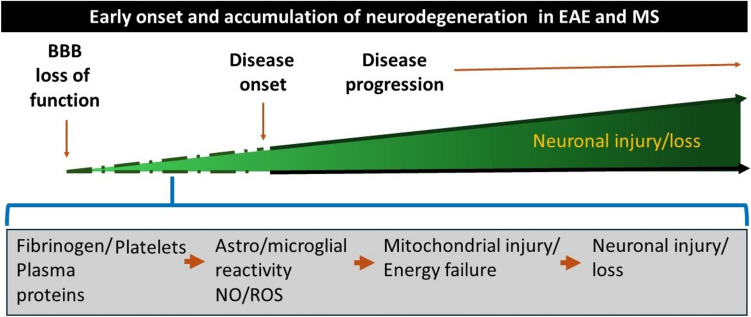

## Introduction

Multiple sclerosis (MS) is an autoimmune disease targeting the central nervous system (CNS) (Woo et al. 2024), affecting over 2.8 million individuals worldwide. The etiology of MS is unknown (Parnell and Booth 2017), but a considerable body of evidence supports the view that it is a multifactorial disease, involving a genetic predisposition and environmental factors such as vitamin D deficiency, viral infections, smoking, early-life obesity (Alfredsson and Olsson 2019) and poor gut microbiome health (Ochoa-Repáraz et al. 2010; Seifert et al. 2018). These combined factors lead to a cascade of events resulting in compromised blood brain barrier (BBB) function, inflammation, demyelination and neurodegeneration (Lassmann 2019).

MS exists as a number of subtypes. Over 80% of affected individuals initially exhibit a relapsing–remitting (RR-MS) clinical profile, typified by episodes of neurological dysfunction interrupted by spontaneous remissions. Within 15 to 25 years and in about 50% of cases, RR-MS transitions to a secondary progressive (SP-MS) form characterized by neurological deterioration without remissions. About 10–15% of total MS cases exhibit a primary progressive (PP-MS) form where neurological decline begins at disease onset. Rare MS forms are also known, such as progressive-relapsing MS where acute relapses are superimposed on the progressive course, or benign MS where patients experience relatively milder symptoms and do not reach an irreversible stage, or paediatric MS beginning before age 16 (Klineova and Lublin 2018). In recent years, the understanding of MS pathophysiology has advanced considerably, highlighting not only the intricacy of pathological processes, but also their evolution over the disease trajectory (Groppa et al. 2021). Thus, from early stage, differential pathological hallmarks are evident in different CNS compartments. White matter (WM) damage is characterized by perivascular lesions associated with severe BBB dysfunction, inflammatory infiltration, demyelination, glial reactivity and axonal injury/loss (Lassmann 2019). These lesions contain numerous T cells, (especially CD8^+^), B cells and macrophages containing myelin debris, associated with axonal injury and severe astrocytic and microglial reactivity. However, while active lesions are the predominant WM hallmark in early disease, other lesion types are also present. This includes chronic active plaques (with low demyelinating activity at the lesion edge and less extensive BBB damage), inactive lesions (showing paucity of macrophage/microglia or T cells), mixed active/inactive and shadow plaques (sharply demarcated areas with reduced myelin density and abnormally thin myelin sheaths). In progressive disease, active lesions are no longer the predominant feature of WM, while slowly expanding lesions are more frequently observed (Filippi et al. 2024). On the other hand, lesions observed in cortical grey matter (GM) regions differ strikingly from those found in WM by exhibiting limited BBB breakdown and reduced T cell and macrophage infiltration relative to WM lesions, but pronounced microglial reactivity and demyelination associated with axonal transections, neuronal apoptosis and reduced neuronal and synaptic density (Bø et al. 2003). Notably, cortical GM lesions are adjacent to severe meningeal inflammation, consisting of diffuse infiltrates or dense cellular aggregates (or follicles) of CD4^+^ and CD8^+^ T cells, macrophages and B and plasma cells (Bø et al. 2003). These lesions develop early in MS and increase in size and number with disease duration.

Processes underpinning progressive disease have been comprehensively reviewed by Papiri et al. (2023) and Filippi et al. (2024). They are associated with worsening neuro/axonal damage and loss across WM and GM, due to chronic inflammation resulting from activated microglia, macrophages, and meningeal lymphoid follicles generating oxidative stress and mitochondrial injury. This, in turn, reduces glia-neuron metabolic support, leading to additional oxidative stress, oligodendrocyte apoptosis, further demyelination, impaired remyelination capacity and axonal degeneration, and ultimately neuronal death. These processes are further exacerbated by underlying ageing mechanisms, such as iron deposition which can cause neuronal death (Papiri et al. 2023) and vascular problems, especially decreased cerebral blood flow which is significantly reduced in GM and associated with dysregulation of neurovascular coupling, the latter showing strong correlation to grey matter atrophy and cognitive decline (Zierfuss et al. 2024).

As an additional mechanism of neurodegeneration, there is accumulating evidence for the role of B cells in MS pathogenesis, not only via antigen presentation to T cells and cytokine release which fuel the inflammatory response, but also in the generation of pathogenic antibodies by plasma cells (Comi et al. 2021). However, the target antigens in MS still remain to be identified. On the other hand, a very recent in vitro study has demonstrated that IgM and IgG antibodies from MS patients’ cerebrospinal fluid (CSF) and sera bound to neuronal derived cell lines, suggesting molecular targets of neuronal origin (Nazir et al. 2023). This study also revealed antibody reactivity against oligodendroglia cell lines, consistent with demyelination as a hallmark of MS and the evidence that B cells produce antibodies against a broad range of CNS antigens (Comi et al. 2021).

Despite years of investigation, factors responsible for early neuronal loss in the cortex in the absence of significant inflammation remains unidentified. There are, however, two consistent observations from post-mortem examinations providing clues as to the nature of the mechanism inducing neurodegeneration (Howell et al. 2014). First, while lymphocytes and plasma cells are confined to the meninges, reactive microglia accumulate at sites of active demyelination. Second, this microglial reactivity co-localizes with regions of active demyelination and neuronal loss, thereby implicating microglial pro-inflammatory mediators in neuronal damage. This had led to the hypothesis that although sequestered, meningeal inflammation represents a source of pro-inflammatory mediators able to infiltrate into the underlying cortex, due to slowing of blood flow in the deeper recesses of the sulci (or furrows on the brain surface) (Lassmann 2019). The presence of these products would thereby trigger a severe microglia reactivity, capable of sustaining a chronic pro-inflammatory environment leading to neurotoxicity. However, the identity of the demyelinating or neurotoxic factors has not been determined to date. Yet, the identification of mechanisms underlying this damage in early disease stage is important, because it carries significant implications for prognosis, patient management and the development of neuroprotective strategies. To elucidate neurodegenerative mechanisms, in particular those relevant to the pre-symptomatic disease stage, investigators have generated a range of animal models of neuroinflammation and demyelination. Based on such approaches, the literature now reports compelling evidence that a new player in inflammation, namely platelets, also represents a major contributing element in neuronal loss.

## Insights Into Inflammatory Demyelination and Neurodegeneration from Animal Models

Unsurprisingly, given the complexity of MS, it has not been possible to generate an accurate animal model for this disease. However, several murine and non-human primate models have been developed, which recapitulate specific facets of MS and have proven extremely informative in terms of elucidating mechanisms underpinning critical aspects of the disease pathophysiology (Procaccini et al. 2015; Kap et al. 2016). These experimental paradigms are generated using a range of approaches including virally, or autoimmune-mediated encephalomyelitis, or alternatively, myelin destruction with the use of toxins.

1. Theiler’s murine encephalomyelitis virus-induced demyelinating disease (TMEV-IDD) represents a valuable tool to examine a potential viral aetiology for MS (Miller and Rodriguez 1995). The virus in delivered via intrathecal injection and induces a chronic disease beginning with an acute encephalomyelitis, followed by a chronic progressive demyelinating phase. Disease severity and course vary depending on TMEV strain, where for example the TMEV DA strain induces severe demyelination in SJL mice, but the TMEV BeAn strain a robust antibody response (Pike et al. 2022). Pathological hallmarks include lymphocytic infiltration, neuro/axonal and oligodendrocyte loss and demyelination, predominantly occurring in periventricular regions and the spinal cord.
2. Toxin-induced demyelination (cuprizone and lysophosphatidylcholine [LPC, also known as lysolecithin]) models exhibit extensive gliosis, microglial reactivity, oligodendrocyte apoptosis and severe demyelination, followed by a remyelination phase. Cuprizone is administered by modification of the diet, resulting in widespread demyelination throughout the WM, cerebral and cerebellar cortical GM and cerebellar peduncles (Blakemore 1973). It is, however, most reproducibly observed in the corpus callosum. The LPS model is generated by a single injection into the spinal cord, resulting in focal demyelination (Hall 1972). Spontaneous and extensive (but usually incomplete) remyelination follows. Therefore, these two models have proven useful in investigations of mechanisms underpinning remyelination and restoration of axon/myelin interactions.
3. Experimental autoimmune encephalomyelitis (EAE) is a model of autoimmune-mediated demyelination induced by immunization with CNS proteins or peptides (Bjelobaba et al. 2018; Glatigny and Bettelli 2018), and is the most widely used model. ‘EAE’ is a generic term to describe multiple models of CNS inflammation in rodents and non-human primates, where each defined antigen/host combination is associated with a unique progression and pathophysiology. Thus, a range of clinical profiles, including monophasic, chronic-progressive, chronic-relapsing EAE have been described. Most protocols make use of an active immunization approach which relies on adjuvants to augment the immunogenicity of the antigen and increase BBB permissiveness, generally resulting in an aggressive disease. However, passive immunization with activated CD4^+^ T lymphocytes does not require adjuvants. EAE recapitulates many MS hallmarks including BBB loss of function, inflammatory infiltration, demyelination, glial reactivity and axonal injury/loss.

EAE variants generated by active immunization in the C57BL/6 mouse with peptide 35–55 of myelin oligodendrocyte glycoprotein (MOG, a quantitatively minor myelin component), or the 1–124 region of the MOG extracellular domain in non-human primates have been particularly favoured, because the MOG protein is a unique antigen in that it can induce both an encephalitogenic T-cell response, as well as a demyelinating autoantibody response (Gold et al. 2006). Also, due to the advent of genetic manipulation techniques the generation of a large array of murine mutants carrying defined lesions has been made possible. These have recently been comprehensively reviewed by Constantinescu et al. (2011), Glatigny and Bettelli (2018) and Dedoni et al. (2023). They range from transgenic models, gene deletion (‘knock-out’), gene insertion (‘knock-in’), tissue-specific and inducible gene deletion (Cre/lox recombination system) models to investigate the functions of defined signalling molecules and their receptors, pathways, the roles of immune subsets and pre-clinical drug evaluation. These approaches have also generated models of spontaneously developing EAE, which have provided insights into mechanisms of disease development, as well as humanized mouse lines to further explore mechanisms of susceptibility. The bulk of examples described below has been derived from murine MOG_35–55_ EAE:

### BBB loss of function

Historically, the EAE model has been the most valuable contributor to the understanding of BBB loss of function and mechanisms promoting molecular and cellular changes in endothelial cells resulting in the generation of the transcellular pathway (Mapunda et al. 2022). Thus, EAE studies have identified CCL2, CCL19, and CCL21 in mediating arrest of immune cells and capture by the intracellular adhesion molecule 1 (ICAM-1) and the vascular cell adhesion molecule 1 (VCAM-1) on the endothelial surface. They have revealed the essential role of caveolae formation, namely the caveolin-rich transmigratory cups that surround the migrating CD4^+^ T cells for transmigration, via the generation of a caveolin-1 gene deletion mutant (Wu et al. 2016). Significantly, they uncovered the unexpected process by which, once arrested, CD4^+^ T cells crawl on inflamed CNS microvessels in search of sites for diapedesis (Engelhardt and Ransohoff 2012). EAE has also allowed investigations of the important immune reactions occurring in the perivascular space. This includes, for example, the early role of perivascular macrophages in leukocyte infiltration, ahead of onset of clinical symptoms (Hoffmann et al. 2002; Polfliet et al. 2002), and of pericytes in facilitating macrophage entry into the CNS via the secretion of ICAM-1 and VCAM-1 and expression of chemokines MCP-1, MIP-1α to promote neuroinflammation (Török et al. 2021; Kaushik et al. 2021). Similarly, EAE is an extensively used model to investigate the complex morphological, transcriptional, biochemical and functional remodelling of perivascular astrocytes in response to the accumulation of inflammatory cells at the vascular surface and subsequent infiltration (Aharoni et al. 2021). More recently, this model has provided insight into the earliest consequences of BBB loss of function associated with components of the coagulation cascade and the complement system (Plantone et al. 2019; Ziliotto et al. 2019).

### Distinct role of cytokine/chemokine networks and contributions of immune subsets

Overall this model has played a leading role in the elucidation of the immensely complex cytokine/chemokine networks, and the signalling pathways responsible for immune cell attraction and migration to the parenchymal side of the BBB (Karpus 2020; Heng et al. 2022). For example, it is now known that upon MOG_35–55_ recognition by antigen-presenting cells (APC), CCR6 downregulation and CCR7 upregulation by APC allow these cells to respond to CCL19 and CCL21 expressed in lymph nodes leading to their migration to T cell rich areas. This leads to IL-6, IL-23, and TGF-ß secretion by APC and differentiation of Th17 cells. CCR7 downregulation by Th17 cells allows egress of these cells from lymph nodes. At the BBB, T cells, monocytes, and neutrophils interact with CCL2 present on the endothelial cell surface via the chemokine receptor 2 (CCR2) expression. Th17 cell and monocyte secretion of IL-17, GM-CSF and TNF promotes lymphocytic extravasation across the endothelial layer, which is potentiated by monocyte and microglial expression of multiple chemokines, including CCL2, CCL3, CCL19, CCL20, CCL21, CCL22, CXCL1, CXCL2, and CXCL10 resulting into lymphocytic accumulation into the CNS parenchyma.

In relation to the above, one of the major contributions of EAE has been in defining T cell subsets, their effector functions and the reciprocal interactions between subsets (Robinson et al. 2014). In particular, the roles of the Th1 and Th17 subtypes have been clarified: while Th1 cells promote cell-mediated responses via the activation of macrophages and other immune cell types, it is evident that Th17 cells are directly responsible for autoimmune-mediated demyelination (Robinson et al. 2014). Much remains to be done, however, in terms of understanding the relationship between Th1 and Th17 cells in initiating/driving pathogenesis, together with the switch to Th2 cells in mitigation of the pro-inflammatory environment and amelioration of clinical disease. In addition, EAE has identified a role for CD8^+^ T cells by the demonstration that myelin-specific CD8^+^ T cells can induce EAE (Sun et al. 2001) and are implicated in active lesions, including via CD8^+^-mediated killing of oligodendrocytes (Saxena et al. 2008). Similarly, EAE has contributed to the understanding of the role of the Treg subset in the maintenance of self-tolerance and in autoimmunity (Robinson et al. 2014).

EAE and gene-targeted mutants have been critical to the functional differentiation between sub-types of mononuclear myeloid cells and their roles under hemostatic or pathological conditions in the CNS (Monaghan et al. 2019). Upon onset of aberrant neuroinflammation, monocytes become the predominant cell sub-type that promotes inflammation by migrating to the CNS via the CCR2 response to chemokine signalling such as CCL2, where they differentiate into APC. CCR2-defcicient mice do not develop EAE (Xu et al. 2021). Perivascular macrophages are believed to modulate immune cell entry (Török et al. 2021; Kaushik et al. 2021). Microglia, on the other hand, do not appear to be major contributors to tissue damage; their role seems to consist rather in controlling inflammation and promoting tissue repair.

With the new appreciation of MS as a B cell-driven disease, studies based on EAE have provided new insights into the role of B cells, which was historically focused on humoral immunity in pathogenesis. It is now evident that B cells have more complex involvement than the generation of oligoclonal IgG, which is a common finding in MS patients and antibody production (Comi et al. 2021). Thus, more recent EAE studies have highlighted the wider effector functions of B cells and B cell subsets in terms of potent antigen presentation to T cells, as well as their involvement in intrathecal antibody production, and the formation of germinal structures in the meninges which exacerbate disease (Comi et al. 2021).

### Mechanisms of neuro/axonal injury and loss

EAE has facilitated the understanding of the multiplicity of destructive mechanisms resulting from infiltration of activated lymphocytes into the CNS, which in addition to effects of autoreactive Th1 and Th17 cells and demyelinating antibodies, culminate into severe axonal and neuronal injury and loss. This includes direct injury from toxic products of macrophages and microglia, namely proteo- and lipolytic enzymes, cytokines, excitotoxins, and reactive oxygen and nitric oxide intermediates (Lassmann 2010). With respect to axonal injury, reactive oxygen species (ROS) and nitric oxide (NO) intermediates are of particular significance as they result in blockage of axonal transport and eventually, irreversible damage. EAE studies have identified that both ROS and NO intermediates impair mitochondrial function resulting in energy deficiency, which is severely detrimental to axons, as well as by increasing their susceptibility to exocytotoxic (glutamate-mediated) injury (Lassmann 2010). EAE studies, and in this case with major inputs from the use of models of toxin-induced demyelination, have also provided valuable insights into the understanding of oligodendrocyte loss and obstacles to remyelination (Zirngibl et al. 2022).

### The relationship between GM pathology and progression

Murine and non-human primate EAE exhibit symptoms that are characteristic of MS, such as visual defects, ambulatory difficulties, problems with balance, bladder and bowel incontinence and pre-onset neuropsychological deficits. Additionally, besides exhibiting WM lesions in the optic nerve, spinal cord, the fimbrium region of the hippocampus and the cerebellum, EAE also has widespread GM pathology in multiple regions affected by MS, for example the retina, hippocampus, thalamus, striatum and the cerebellum. Lesions exhibit hallmarks of one sub-type of MS lesions (Lucchinetti et al. 2000). The cerebellum in particular, has been a region of interest for comprehensive investigations aimed at validating EAE for understanding GM MS pathology (Voskuhl and MacKenzie-Graham 2022). These investigations revealed diffuse inflammatory infiltration in all cortical layers associated with demyelination, prominent microglial reactivity, swollen axons and axon retraction bulbs, all of which are hallmarks of MS GM pathology. Loss of Purkinje neurons and disorganized arborization of Purkinje neurons were also documented. MRI studies revealed decreased global cerebellar volume, which showed an inverse relationship with disease duration and corelated with GM volume reduction, but not WM volume loss. Cerebellar GM atrophy also strongly correlated with worsening clinical scores. (Voskuhl and MacKenzie-Graham 2022; Hamilton et al. 2019).

### The relationship between gut dysbiosis and MS

Due to the identification of poor gut microbiome health as a risk factor for MS, the significance of the gut-brain axis in health and disease is a subject of increasing interest, and evidence based on the EAE model strongly suggests a relationship between gut microbiota and MS development. The strong body of evidence from preclinical and clinical investigations have been reviewed by Forbes et al. (2016). These studies include dietary interventions, where intermittent fasting, or calorie restriction regimens prior to EAE induction mitigated disease aggressiveness (Purdel et al. 2023). Faecal transfer studies from mice housed in a conventional environment to recipient mice maintained in a specific pathogen-free environment produced a more aggressive form of EAE in recipient mice (Wang et al. 2021). In other studies, oral administration of a cocktail of antibiotics prior to EAE induction (Ochoa-Repáraz et al. 2010; Seifert et al. 2018) had the effect of significantly reducing the gut bacterial population, decreasing levels of pro-inflammatory cytokines and increasing anti-inflammatory counterparts, including IL-10, IL-13, together with the Foxp3^+^ Treg population. This was associated with a reduction in the proportion of mice exhibiting disease development and overall disease severity. Clinical trial validation of the beneficial effects of gut microbiota modulation is still unavailable, but to date, evidence from EAE studies show promise for the modulation of gut dysbiosis as an adjunct therapy for the control of inflammation.

### Potential of non-coding RNA as therapeutic targets, or biomarkers

In recent years, evidence has accumulated for a role for multiple forms of non-coding RNAs, but microRNAs (miRNAs) in particular, as gene expression regulators and their potential as therapeutic targets, or biomarkers for MS (Cipriano et al. 2024). The rationale behind this concept is that CNS injuries occurring during MS pathogenesis have been shown to correlate with miRNA dysregulation, thereby influencing activation, differentiation, together with T and B cell function, and contributing to disease development and progression.

For example, miR-326 was found to be overexpressed in Th17 cells that produce IL-17 in RR-MS patients (Du et al. 2009), while miR-590 was overexpressed during relapse (Liu et al. 2017) and, notably, B cell expression of miR-326 and miR-155 promote differentiation into antibody producing plasma cells (Du et al. 2009). The pro-inflammatory miR-155 is strongly upregulated in the serum and in CNS lesions of MS patients and accordingly, miR-155 deletion resulted in reduced numbers of infiltrating Th17 T cells in EAE (Thompson et al. 2023).

A potential therapeutic role of miR-145-5p was also revealed through EAE studies (Kornfeld et al. 2021). This component is highly expressed in oligodendrocyte progenitor cells (OPC), but downregulated during their differentiation into myelinating oligodendrocytes, concurrently with the myelin gene regulatory factor (MYRF). MiRNA-145-5p gene deletion leads to spontaneous differentiation of OPCs, suggesting a potential therapeutic application for remyelination in MS.

An analysis of serum miRNAs was performed over a four-year follow-up study, in 73 RR- and PP-MS focussed on miR-191-5p, miR-128-3p, miR-24-3p, and miR-223-3p, based on prior evidence of association of these miRNA species with MS (Vistbakka et al 2022). Over this period, expression of miR-128-3p and miR-24-3p was stable longitudinally, while temporal changes of miR-191-5p and miR-223-3p were observed. Temporal changes in miR-191-5p were observed to be associated with increases in disability, but without statistical difference between MS subtypes, while the variability of miR-223-3p was associated with relapses, thereby suggesting MiR-191-5p as a significant biomarker of progression. The differential expression of miRNAs in various biological compartments and fluids, such as CSF, serum, and PBMCs, is an area of interest for the discovery of new biomarkers of progression which will be heavily dependent upon the use of gene deletions to silence specific miRNAs (Cipriano et al. 2024).

In summary, murine MOG_35–55_ EAE has been invaluable in the development of a conceptual framework for the roles of immune cells, their pathological signatures and modulation of their phenotypes during the acute phase and disease resolution. Additionally, the model has facilitated the understanding of the array of downstream mechanisms via which autoreactive cells and their products induce demyelination and axonal injury. Recent studies also highlight the potential validity of the model to investigate risk factors and identify biomarkers. Furthermore, it is of significance that major hallmarks of MS GM pathology, namely absence of lymphocytic infiltration, neuronal loss, elevated microglial reactivity, presence of follicles in meninges and the relationship between GM atrophy and progression, are clearly evident in EAE (Voskuhl and MacKenzie-Graham 2022; Orian and Maxwell 2023). Therefore, overall, EAE recapitulates the differential inflammatory processes taking place in the CNS during MS, whereby WM disease is characterized by overt BBB loss of function and inflammatory lesions and GM by neurodegeneration in the presence of less significant inflammation, but major brain volume loss. The sum of these data, therefore, strongly validate EAE for generating proof-of-concept for the core events which initiate and promote neurodegeneration, and which cannot be directly addressed in the human counterpart of the disease, because these events occur during the pre-symptomatic disease stage. Additionally, a major advantage provided by any animal model is the ability to select defined time windows for investigations of mechanisms underlying specific stages of the initiation and progress of pathology. Murine EAE, induced both actively and by adoptive transfer, exhibits relatively synchronous disease onset (Orian et al. 2024), whereby emergence of clinical signs ranges over a 4–5 day time span within a given cohort of experimental animals, as opposed to non-human primate EAE where onset can vary between 2 to 16 weeks ('t Hart et al. 2000). This model, therefore, is perfectly suited for examination of the molecular and immunopathological sequence of events preceding overt disease. Our laboratory has focused on the pre-onset stage of disease, to obtain clues as to the basis of neurodegeneration and identified an early and platelet-driven neuroinflammation and a concomitant direct attack on neurons.

## The Role of Platelets in Neuroinflammation

Platelets (also known as thrombocytes) are blood cells originating from megakaryocytes, which have been studied for decades in the context of blood clotting and vascular haemostasis (Rondina and Garraud 2014; Orian et al. 2021; Nicolai et al. 2024). They are anucleate cells of 1.5–3 µm in diameter in humans, characterized by their abundance (150–450 × 10^3^/µl) and short lifespan (8–9 days) (Kapur et al. 2015). The corresponding values for mice are 0.5–1.0 µm in diameter, 1,000–1,500 × 10^3^/µl and a half-life of 4–5 days (Wassmer et al. 2021). However, due to a large part in the advent of profiling techniques, it has emerged that these cells should be viewed as immune cells with functions beyond their classical ones. They express cytokine and cytokine receptors (Power et al. 1995), immune cell signature molecules such as toll-like receptors (TLR) and NOD-like receptors (NLR) (Rondina and Garraud 2014; Koupenova et al. 2022). TLRs in particular, play a vital role in immunity since they link the innate immune system, the body’s first line of defence against pathogens, with the adaptive immune system, a more specific response against pathogens. Platelets also contribute to the recruitment of other immune cells, such as monocytes, neutrophils and macrophages (Gros et al. 2015). These features underline their link with inflammation, tissue repair, autoimmunity and possibly more functions.

Despite the absence of nucleus, these cells have a complete range of RNA species, a large megakaryocyte-derived mRNA reservoir (including pre-mRNA) containing an estimated 3,000–6,000 unique transcripts which are specifically sorted rather than randomly transferred during thrombopoiesis (O’Neill et al. 2000; McRedmond et al. 2004). Platelet mRNA is of high stability, provided by a cap structure at the 5’ end and a ploy-A tail. Platelets possess a functional spliceosome, allowing for rapid adaptations to changing environmental cues and a change of phenotype (Smyth et al. 2009). Also identified in platelets are ribosomal RNA (rRNA), microRNAs (miRNAs), small nuclear (snRNA), small nucleolar RNA (snoRNA), antisense RNAs (asRNA), and transfer RNA (tRNA) (Weyrich and Zimmermann 2004). The platelet miRNA population includes at least 284 unique species which regulate protein expression via miRNA-mRNA pairings (Nagalla et al. 2011). The complex array of RNA species within platelets and their impact on platelet biogenesis and platelet function have been further reviewed by Neu et al. (2020).

Platelets carry rough endoplasmic reticulum and polyribosomes and are capable of protein synthesis and regulation of expression. It is estimated that platelets are capable of synthesizing up to 3,700 proteins (Wachowicz et al. 2016). These relate not only to hemostasis as expected, namely coagulation factors and receptors involved in thrombosis, membranes, receptors, ion channels, adhesion molecules, but also to defence, cell–cell communication, chemokines, cytokines, signal transduction, growth factors, enzymes involved in the synthesis of lipid signalling molecules and others. These components are stored in their cytoplasm or in different types of subcellular compartments, in processed, or precursor forms and can be released instantly upon activation signals. This profile provides compelling evidence for platelets as effector cells in inflammation and immune modulation.

Protein content can also be augmented by their ability to scavenge proteins from their environment, such as albumin, fibrinogen and neurotransmitters from the circulation (Canobbio 2019), accounting for a substantial proportion (up to 30%) of platelet protein content. They take up neuronal proteins, in a way which reflects the disease of interest. It is not clear how this occurs and what might be the significance of this phenomenon. This has led to the concept that platelets can be ‘educated’, namely acquire a unique mRNA and protein profile, depending on pathological status.

The range of platelet functions increases rapidly in the literature. It has been estimated that only 10% of circulating platelets actually participate in vascular hemostasis (Burnouf and Walker 2022), demonstrating that such functions need to be viewed in the context of their broader role as immune cells. Under hemostatic conditions they can cross the BBB, via an unknown transport mechanism. There is evidence that they form part of the neurovascular unit (Burnouf and Walker 2022), which suggests a protective role for these cells.

**Fig. 1:**
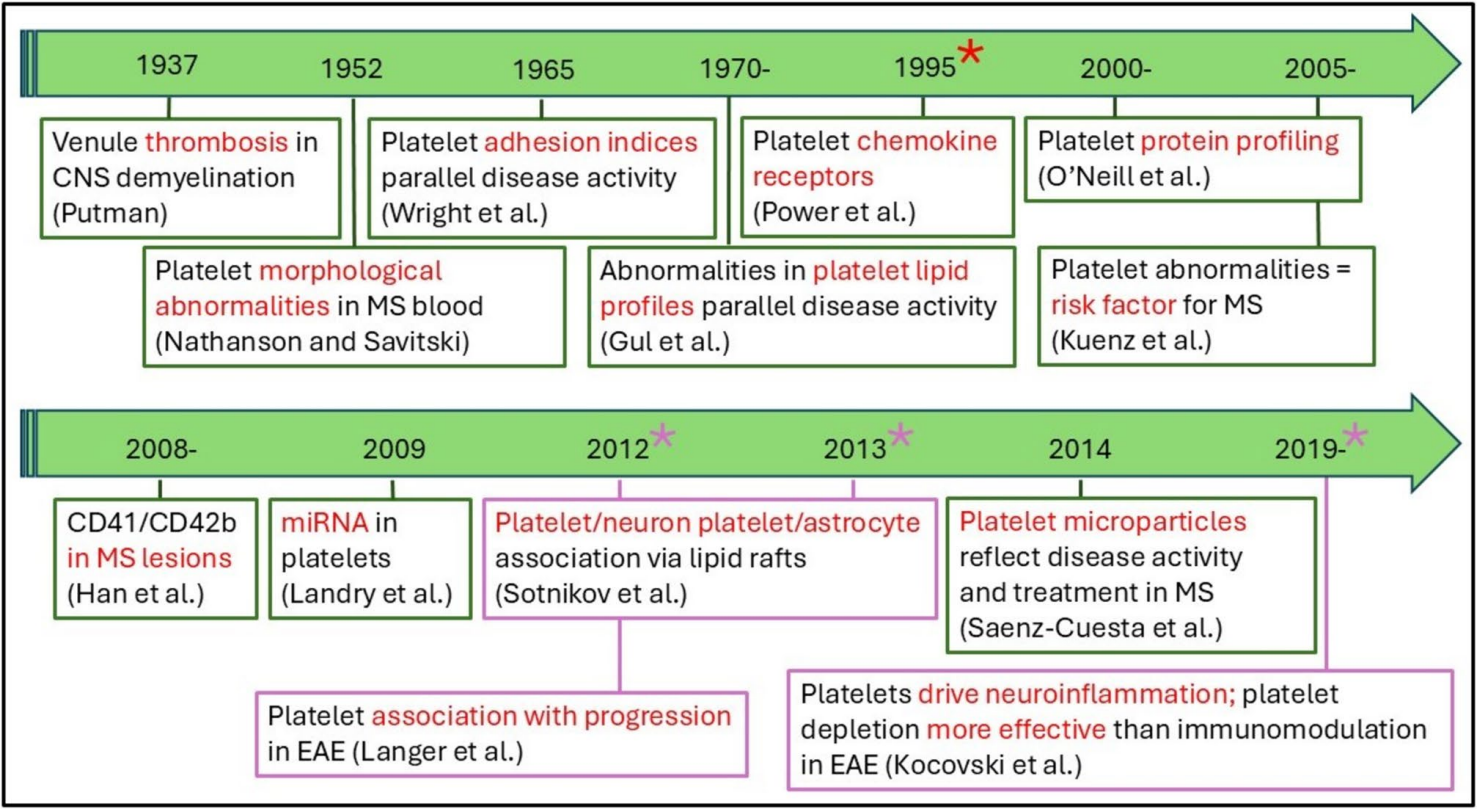
Association between platelet abnormalities and MS. The timeline shows a number of key findings supporting the concept of a critical role for platelets in the development of MS, beginning with the first evidence of thrombi in MS lesions. The identification of chemokine receptors on platelets (*) provided evidence that platelets are immune cells. More recently, investigations in animal models, principally EAE (* and purple boxes), have allowed the demonstration of the role of platelets in driving disease development and of the detrimental effects of platelet associations with CNS elements. Complete citations are shown in the reference list

There has long been an association between platelets and MS (Fig. 1), which has been reviewed by Orian et al. (2021) It began with the observation of venule thrombosis in CNS demyelination (Putman 1937). Subsequently, evidence was presented of altered platelet adhesiveness and ultrastructure (Nathanson and Savitski 1952; Wright et al. 1965), together with biochemical abnormalities, especially in lipid composition of platelet membranes, and by the presence of membrane-bound components which regulate mechanisms modulating platelet aggregation (Gul et al. 1970), all of which are indicators of platelet activation. In disease, evidence for platelet-specific markers have been detected in the plasma of MS patients and in MS acute, chronic active and chronic plaques, namely glycoproteins IIb and IIIa (Lock et al. 2002). Platelets are also high extracellular vesicles (EV) producers and platelet microvesicles are the most abundant microparticle population in peripheral blood, accounting for around 70–90% of the total microvesicle population (Saenz-Cuesta et al. 2014). However, with respect to a relationship between MS sub-type and platelet microparticle levels there is incomplete agreement in the literature. Marcos-Ramiro et al. (2014) uncovered significantly increased platelet microparticles relative to healthy controls in RR-, SP- and PP-MS cases, namely in all clinically definite MS forms, but not in clinically isolated syndrome individuals, thereby suggesting that that platelet dysfunction manifests when patients are definitely progressing. On the other hand Saenz-Cuesta et al. (2014) demonstrated no significant difference in SP-MS, but elevated microparticles in RR-MS individuals and a relationship between numbers of circulating microparticles and disease status. This is in contradiction with recent evidence from Morel et al. (2015) demonstrating significant platelet activation in SP-MS, although changes, if any, in actual platelet microparticle numbers were not reported in this study. Therefore, the potential of platelets/platelet microparticles as biomarkers is yet to be determined. Finally, studies have revealed the close interplay between platelets and the coagulation system, as well as with the complement system during neuroinflammation (Ziliotto et al 2019). Indeed, markers of the coagulation cascade have been identified in MS lesions (Han et al. 2008) ahead of demyelination (Plantone et al. 2019), suggesting that coagulation factors infiltrate CNS tissue from the acute phase of the disease, triggering microglial reactivity. The cytotoxicity of these products, besides initiating an inflammatory response, may lead to neurodegeneration.

**Fig. 2:**
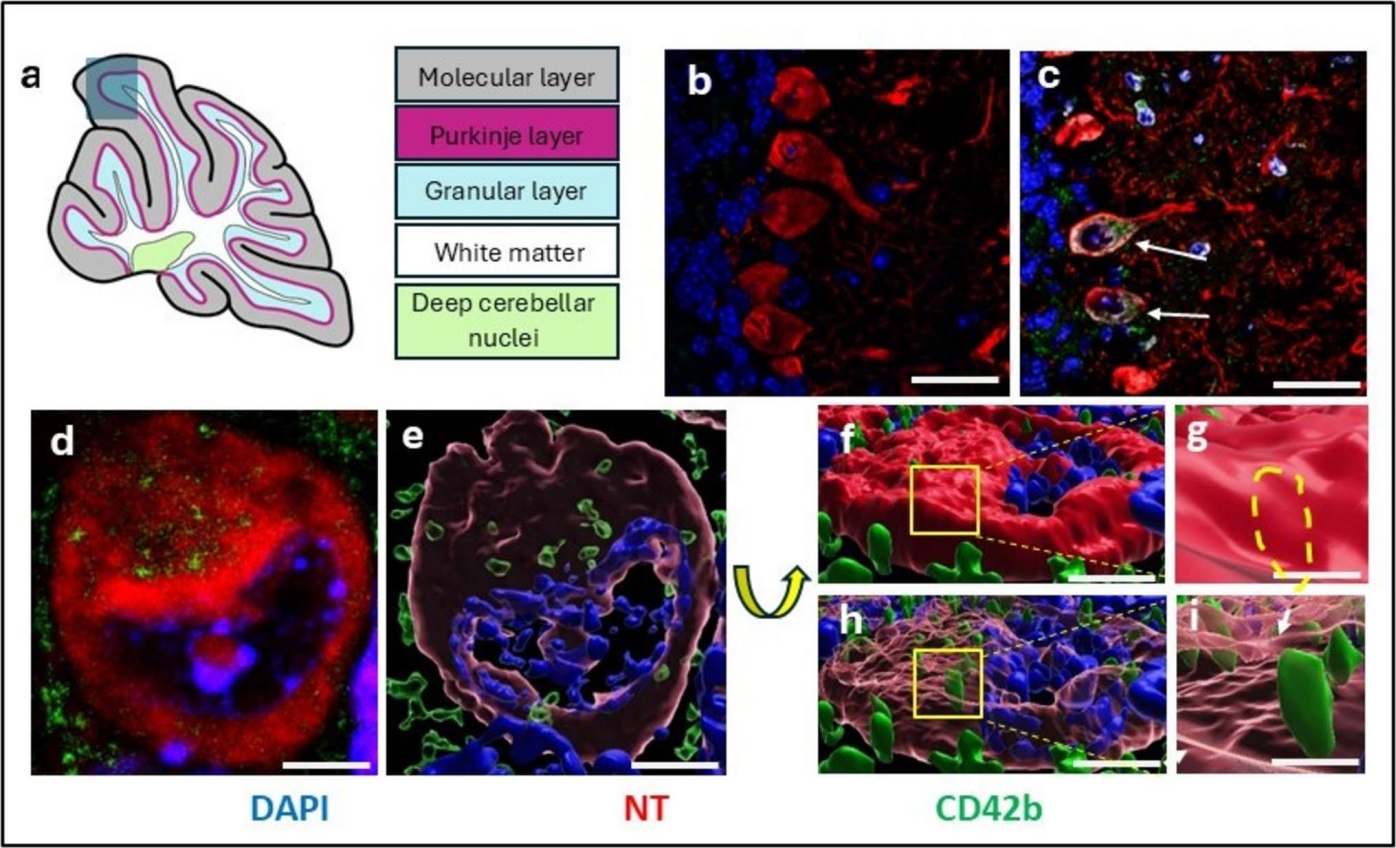
Platelet-neuron association and platelet internalisation by neurons**.** From early EAE, platelets infiltrate the CNS and associate with neurons. Cerebellar tissue from MOG_35–55_-EAE-induced C57BL/6 mice was taken at 5 days post disease induction, equivalent to a clinical score of zero. Sections, cut at 30 µm, were immunostained with anti-CD42b to identify platelets (green), and NeuroTrace to identify neurons (**NT**; Invitrogen™ NeuroTrace™ 640/660 Deep-Red Fluorescent Nissl Stain); nuclei were stained with DAPI (blue). **a** representative diagram of section at the midline, showing the anatomical structure of the cerebellum, with the region of interest (Purkinje layer) shaded in grey; (b) representative section from a sham-injected control mouse, showing absence of platelets, in contrast with (c) taken from a MOG_35–55_-EAE-induced mouse showing platelet-neuron associations (white arrows) (d) magnified image of a Purkinje cell from (c) and (e) IMARIS analysis showing a transparent image of the same cell demonstrating intimate associations between platelets and neurons. Rotation of the image in (e) and comparison between the non-transparent (f and g, external aspects) and transparent (h and i, internal aspects) IMARIS representations, confirm that platelets are internalized by neurons. Small arrow in f shows the neuronal cell membrane. Scale bar = 100 µm in b-c and 20 µm in d-i

An association between platelets and EAE development was first demonstrated by Langer et al. (2012), when mice were treated with platelet antibodies during the acute phase of the disease, resulting in amelioration of clinical profile. Our laboratory subsequently identified the early and significant platelet accumulation in the circulation from three days post immunization (Sonia D’Souza et al. 2018). We also established that platelet accumulation occurred clearly ahead of that of autoreactive T cells, and that blocking platelets in the late pre-symptomatic stage resulted in elimination of EAE (Sonia D’Souza et al. 2018; Kocovski et al. 2019). These data support a driving, rather than exacerbating role for platelets in disease development. Furthermore, we showed evidence of platelet-neuron aggregates in multiple regions along the neuraxis, together with platelet internalization by neurons (Fig. 2), from the pre-onset stage (Sonia D’Souza et al. 2018; Kocovski et al. 2019 and 2021). This includes the hippocampus where we demonstrated that these associations were linked to significant anxiety-like behaviour (Kocovski et al. 2019 and 2021). The identification of the platelet-specific pro-inflammatory marker PF4 around neurons suggests that this interaction is pathological rather than protective (Sonia D’Souza et al. 2018). In support of this notion, evidence from ex vivo studies strongly suggest that platelet secretions can be neurotoxic. This was demonstrated by Joseph et al. (1992a and 1992b) using newborn rat spinal cord explants exposed to purified rat or human platelets stimulated with collagen or thrombin, both of which are major platelet activators in vivo, in the range that would normally be present in a unit volume of whole blood before and after platelet activation. Following a 72-h incubation, a 70% reduction in surviving neurons in the presence of stimulated platelets relative to unstimulated platelets, or red blood cells, as controls. Altogether, these data suggest that from the earliest stages of disease development, platelets contribute to the generation of a highly pro-inflammatory environment, with consequences for neuronal survival. The observation of platelet-neuron aggregation concurs with a separate study, which also revealed that these associations are mediated via lipid-rafts (Sotnikov et al. 2013). In the latter study, platelet-astrocyte interaction was also demonstrated, as well as in a separate study based on the APP-PS1 transgenic mouse, characterized by severe amyloid plaque formation in the absence of cerebral amyloid angiopathy (Kniewallner et al. 2020). It is also possible that platelets associate with myelin or axons, from observation of the immunostaining pattern on the spinal cord, shown by Langer et al. (2012) which is typical of WM tracts.

Additionally, evidence comparing different disease stages, or different disease models further illustrate the complex role of platelets in neuroinflammation. A study by Starossom et al. (2015) comparing early and late EAE showed that in early-stage platelet pro-inflammatory products serotonin (5HT), PF4 and PAF contributed to the priming and activation of myelin-specific T cells in vivo and their differentiation towards the Th1 and Th17 phenotypes. However in late-stage platelets production of pro-inflammatory molecules and T cell stimulation weakened, but these cells exhibited enhanced ability to form aggregates with T cells. In separate studies, using the TMEV-IDD model, Ahmad et al. (2024) showed that platelets play a role in TMEV infection by modulation of expression of immune-related genes, viral clearance and anti-viral antibody isotype response. In the LPS model, examination of the remyelinating phase showed platelet aggregation in the vicinity of demyelinated regions, which was modulated by platelet blocking with anti-CD42b (Philp et al. 2024). Taken together, the evidence of platelet involvement in neuroinflammation in multiple disease models supports the concept of a critical role for these cells.

## How do Platelets Cause Neurodegeneration in Ms?

Given the accumulation of evidence for platelet as immune cells and for their early and critical role in the development of neuroinflammation, a relationship between platelets and MS/EAE, should come as no surprise. Similarly, the extravasation of these cells into the CNS parenchyma is also explicable, as like immune cells in general, they would both generate and respond to signalling molecules and migrate to the target area. There is evidence in the literature that under neurodegenerative/neuroinflammatory conditions, such as Alzheimer’s, disease, neurotrauma, sub-arachnoid haemorrhage and chronic hypertension (Kniewallner et al. 2020; Leiter and Walker 2020) platelets acquire the ability for large scale infiltration of the CNS. Therefore, the key questions are: what are the primary events that attract platelets across the BBB? Why do they apparently specifically associate with astrocytes and neurons? Do these associations lead to lethal consequences? Here, we propose a scenario mainly based on studies of the EAE model, as well as evidence from the literature.

### Primary events that attract platelets across the BBB

**Fig. 3:**
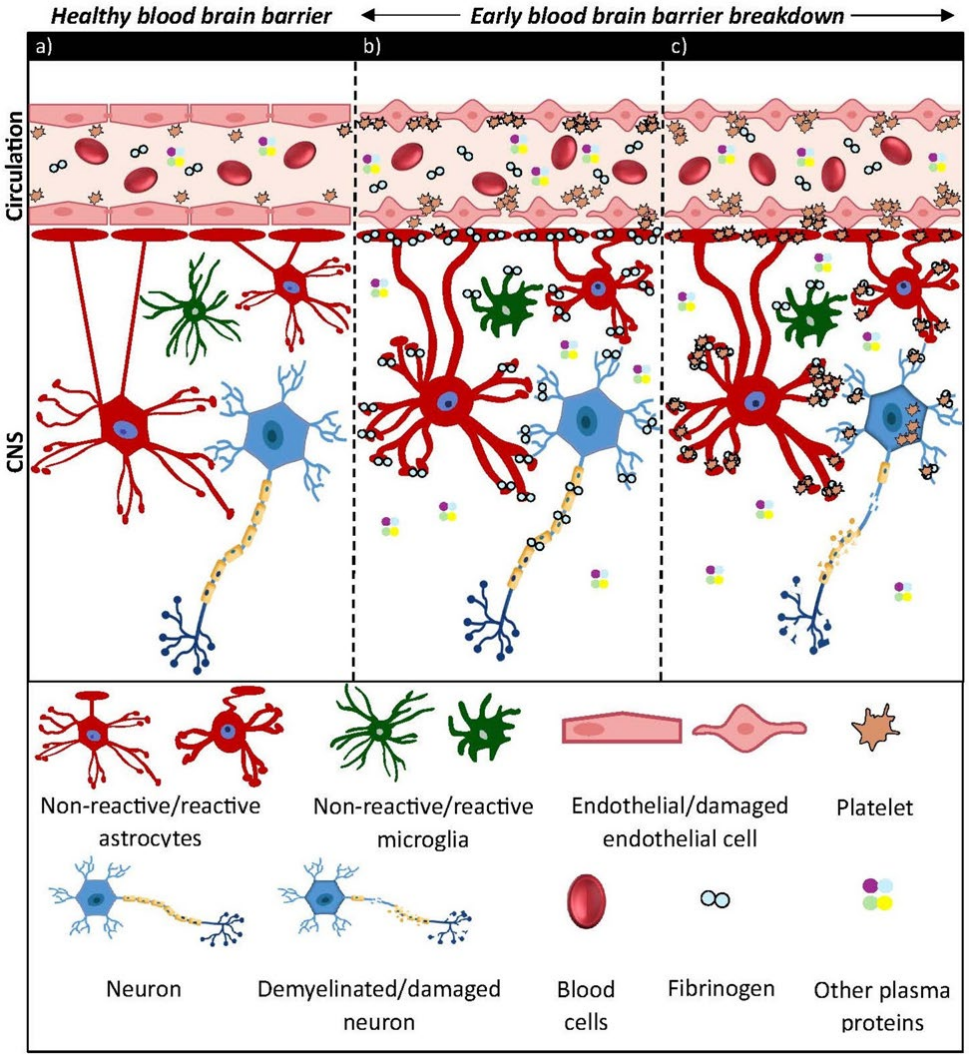
Schematic representation of BBB loss of function and fibrinogen-driven platelet infiltration in the CNS parenchyma. **a** Under steady-state conditions platelets circulate along the blood vessel walls in the presence of an intact BBB. **b** Upon disease onset, damage to the cerebral endothelia layer is associated with leakage of plasma proteins, including fibrinogen, together with fibrinogen binding to glial and neuronal cells and platelet activation and aggregation on endothelial cells. **c** The presence of fibrinogen in the CNS parenchyma acts as a signal for platelet infiltration and association with astrocytes and neurons

The most likely signal that would initiate platelet involvement in EAE/MS pathophysiology is a disruption of BBB integrity. Because of the high responsiveness of platelets to changes on the vascular surface, this would be associated with immediate platelet accumulation on the BBB surface. Disruption of BBB integrity leads to abundant extravasation of plasma proteins, including coagulation cascade factors, notably fibrinogen the major plasma coagulation factor, as demonstrated in MS and EAE, other neurodegenerative diseases and traumatic injury (Davalos et al. 2019; Ryu et al. 2018; Saluk-Bijak et al. 2019). Fibrinogen is synthesized in the liver, and is present in blood as a 340KD glycoprotein, circulating as a homodimer at a concentration of around 2 g/l. Upon activation of the coagulation cascade, fibrinogen levels are upregulated several fold and the protein is converted to insoluble fibrin by the enzyme thrombin, which results in exposing polymerization sites facilitating platelet aggregation and clotting (Koudriavtseva et al. 2020). The presence of fibrinogen in MS plaques has been documented (Lock et al. 2002) and inhibition of fibrinogen has been proposed as a therapeutic approach for MS (Davalos et al. 2019). In support of these observations, a number of studies in animal models demonstrate fibrin deposition prior to the earliest detectable T cell infiltration, or alternatively, the beneficial effects of inhibiting fibrinogen in the EAE model (Ryu et al. 2018). However, fibrinogen possesses functions other than blood clotting, including inflammation, tissue repair and cancer development (Petersen et al. 2018). In CNS disorders, fibrinogen is known to have multiple detrimental effects, such as microglial activation, axonal injury, inhibition of oligodendrocyte lineage cell differentiation and maturation, which lead to impairment of remyelination, as well as opening of the BBB via direct interactions with endothelial cells (Ziliotto et al. 2019). Therefore, it is conceivable that the combination of platelet accumulation on the BBB surface, increased BBB permeability, leakage of fibrinogen into the parenchyma, together with microglial activation and concomitant release of chemoattractants is the driving force that promotes platelet infiltration into the CNS parenchyma, as an early event (Fig. 3). In EAE our data show that this is already evident by 5 days post disease induction, while autoreactive T cells only become first detectable from 10 days onwards (Kocovski et al. 2019).

### Platelet association with glial cells and neurons

There is considerable evidence in the literature for interactions between fibrinogen and CNS elements in CNS disease, which exacerbate inflammation. Fibrinogen has the capacity to bind to extracellular receptors on astrocytes (Wen and Zhang 2023), including I-CAM 1 and the prion protein PrPc (a cell surface, glycosylphosphatidylinositol-anchored glycoprotein), which has been shown to result in upregulation of pro-inflammatory molecules and production of ROS and NO, both of which lead to oxidative stress and axonal injury, as mentioned earlier. Fibrinogen also acts as directly on neurons leading to upregulation of pro-inflammatory cytokines, activation of NF-κB transcription factor and exacerbation of neuroinflammation (Sulimai and Lominadze 2021), in addition to causing mitochondrial injury and neuronal cell death via ROS and NO production (Ziliotto et al. 2019). Finally, there is evidence for direct binding of fibrinogen to microglia, initiating a signalling cascade and a microglial change in phenotype to a phagocytic state. In summary, fibrinogen has effects on multiple CNS cell-types, causing the ROS and NO accumulation and oxidative stress and consequentially in neuronal apoptosis.

Apart from the direct detrimental effects of fibrinogen on neurons and glial cells, the evidence indicates that the association of fibrinogen with astrocytes and neurons can result as an attractant for platelets (Sotnikov et al. 2013). Trauma, infection and inflammation are factors which disrupt the structure of the neurovascular unit, resulting in the exposure of ganglioside-rich lipid rafts which contain ligands recognized by platelets. The implication is that following fibrinogen leakage into the parenchyma and in response to pro-inflammatory mediators of microglial origin, platelets then cross the BBB and become specifically associated with astrocytes and neurons via ganglioside-rich lipid rafts. The demonstration of platelet-specific pro-inflammatory mediators in early EAE suggests that these associations are detrimental to neuronal survival.

### Consequences of platelet-astrocyte and platelet-neuron associations

Platelets entering the CNS parenchyma are highly reactive as demonstrated by the identification of platelet-specific pro-inflammatory molecules in CNS tissue of EAE mice (Sotnikov et al. 2013; Sonia D’Souza et al. 2018), which would contribute to the overall pro-inflammatory environment and oxidative stress, but the consequences of their subsequent associations with neuron and astrocytes are undescribed in the literature. With respect to astrocytes, prominent astrogliosis, which is characterized by proliferation (or at least enhanced detectability) and hypertrophy, is a well-documented response and indeed has been reported both at the BBB and throughout the parenchyma by this laboratory (Pham et al. 2008). This implies that platelet-astrocyte associations do not have an overall detrimental effect on the astrocyte population. On the other hand, given the early timing and extent on neuronal loss, together with the accumulation of platelet-specific pro-inflammatory molecules such as PF4 around neurons (Sonia D’Souza et al. 2018), it would appear that on balance platelet-neuron associations are not compatible with neuronal survival. This disparity may be explained by the differential capacity of these two cell types to withstand damage and injury, whereby astrocytes have highly robust antioxidant abilities and consequently, are more resistant to damage and impaired energy supply than neurons (Kim et al. 2019). Additionally, while we have demonstrated platelet internalization by neurons, we have never observed a similar phenomenon in astrocytes.

## Discussion

The current consensus is that neuro/axonal damage is an early hallmark of MS and a major contributor to disease progression and permanent disability, hence the importance of identifying the key elements in this process, the timing of their involvement and their interactions, for the purposes of developing effective neuroprotective strategies. Based on the above evidence, where EAE studies and immunopathological analyses have played prominent roles, there is reason to believe that platelets play a significant role in this damage from disease onset. This hypothesis is based on two lines of evidence. The first relates to events associated with BBB loss of function, which have been shown to result in exposure of glial cells and neurons to the direct and harmful effects of fibrinogen and other plasma proteins, and concomitent generation of an environment conducive to oxidative stress. Here we suggest that this inflow of fibrinogen represents the signal for early platelet infiltration into the CNS parenchyma and that the pro-inflammatory state of platelets would significantly amplify the effects of plasma proteins and microglia. This process is relevant to both WM and GM.

The second process is based on our evidence of platelet internalization by neurons from the pre-symptomatic stage. While the BBB becomes permissive to large-scale infiltration in WM, the same is not observed in cortical GM. Instead, in GM BBB permissiveness remains limited, but GM becomes exposed to infiltrating soluble, toxic products from lymphocytes confined to germinal centres in the meninges. We propose that the limited BBB loss of function in GM is sufficient to allow platelet extravasation, which also contributes to the severe microglial reactivity observed in cortical lesions.

The extent to which these processes would generate a challenging environment for neuronal survival and the timing of significant irreversible neuronal damage have yet to be demonstrated. However, the existing experimental paradigms can provide valuable proof of concept to validate this hypothesis, for example in vivo studies of fibrinogen inhibition in the EAE model beginning from the pre-symptomatic stage, or in vitro studies evaluating the effects of platelet-rich plasma from EAE or sham-injected mice on primary neuronal cultures. Additionally, due to the limitations on EAE, namely the fact that EAE only partially recapitulates MS, the use of additional models also characterized by BBB breakdown, would enable further investigation of a cause-and-effect relationship between fibrinogen extravasation and platelet infiltration of the CNS. This includes for instance the cuprizone model, where BBB permeability (albeit relatively mild), in the absence of inflammatory infiltration is a pathological hallmark in common with EAE (Berghoff et al. 2017). Additionally, this relationship could be verified using TMEV-IDD model, which reproduces major MS hallmarks, similarly to EAE, but via a different pathogenetic mechanism.

With disease progression, the accumulation of oxidative stress together with additional mechanisms discussed elsewhere (Lassmann et al. 2012), including mitochondrial DNA deletions, iron accumulation with ageing and antibody attack, continue to drive the demise of neurons. Therefore, the sum of the evidence portrays a scenario where, from the earliest point when the BBB function becomes compromised and throughout the disease course, neurons are subjected to an accumulation of destructive mechanisms, leading eventually to transition to the progressive stage of MS.

This role for platelets is compatible with multiple key observations pertaining to MS pathology. These include to begin with, the presence of thrombi and platelet-specific markers in acute lesions, as well as the early occurrence of cortical neuronal loss from the pre-symptomatic stage and that this can take place in the presence of limited lymphocytic infiltration. It is also compatible with the evidence of neurofilament protein in the CSF as a marker of MS. It suggests a downstream mechanism for the now well-established evidence of infiltration of components of the coagulation cascade and of abundant fibrin/fibrinogen in the cortex.

## Conclusions

Taken together, therefore, the evidence in the literature supports the notion that platelets represent an early and active participant in neurodegeneration. This has implications for the development of novel neuroprotective therapies for MS directed at limiting platelet reactivity. Such therapeutics are already being advocated for neurodegenerative (Beura et al. 2023) disorders where platelet hyperactivity and hyperaggregability are viewed as critical risk factors for developing vascular dysfunctions, including Alzheimer's disease, Parkinson's disease and Huntington's disease. The challenge in the use of these therapeutic strategies in patients with MS would be the increased risk of bleeding complications. The use of agents designed to prevent platelet aggregation can carry a risk of intracranial haemorrhage, or gastrointestinal bleeding or skin bruising. On the other hand, the quest for safer drugs is ongoing (Navarro et al. 2024) and there is considerable clinical expertise in the use of such drugs and the management of their long-term use in patients (Swan et al. 2020). In conclusion we propose that platelet targeting as a neuroprotective strategy for MS warrants further investigation.

## Data Availability

No datasets were generated or analysed during the current study.

## References

[CR1] Aharoni R, Eilam R, Arnon R (2021) Astrocytes in multiple sclerosis—Essential constituents with diverse multifaceted functions. Int J Mol Sci 22:5904. 10.3390/ijms22115904 34072790 10.3390/ijms22115904 PMC8198285

[CR2] Ahmad I, Omura S, Sato F, Park AM, Khadka S, Gavins FNE et al (2024) Exploring the role of platelets in virus-induced inflammatory demyelinating disease and myocarditis. Int J Mol Sci 25:3460. 10.3390/ijms25063460 38542433 10.3390/ijms25063460 PMC10970283

[CR3] Alfredsson L, Olsson T (2019) Lifestyle and environmental factors in multiple sclerosis. Cold Spring Harb Perspect Med 9:a028944. 10.1101/cshperspect.a028944 29735578 10.1101/cshperspect.a028944 PMC6444694

[CR4] Berghoff SA, Düking T, Spieth L, Winchenbach J, Stumpf SK, Gerndt N et al (2017) (2017) Blood-brain barrier hyperpermeability precedes demyelination in the cuprizone model. Acta Neuropathol Commun 5:94. 10.1186/s40478-017-0497-6 29195512 10.1186/s40478-017-0497-6 PMC5710130

[CR5] Beura SK, Dhapola R, Panigrahi AR, Yadav P, Kumar R, Reddy DH (2023) Antiplatelet drugs: Potential therapeutic options for the management of neurodegenerative diseases. Med Res Rev 43:1835–1877. 10.1002/med.21965 37132460 10.1002/med.21965

[CR6] Bjelobaba I, Begovic-Kupresanin V, Pekovic S, Lavrnja I (2018) Animal models of multiple sclerosis: Focus on experimental autoimmune encephalomyelitis. J Neurosci Res 96:1021–1042. 10.1002/jnr.24224 29446144 10.1002/jnr.24224

[CR7] Blakemore WF (1973) Remyelination of the superior cerebellar peduncle in the mouse following demyelination induced by feeding cuprizone. J Neurol Sci 20:73–83 4744512 10.1016/0022-510x(73)90119-6

[CR8] Bø L, Vedeler CA, Nyland HI, Trapp BD, Mork SV (2003) Subpial demyelination in the cerebral cortex of multiple sclerosis patients. J Neuropathol Exp Neurol 62:723–732. 10.1093/jnen/62.7.723 12901699 10.1093/jnen/62.7.723

[CR9] Burnouf T, Walker TL (2022) Platelets and brain function. Blood 140:8. 10.1182/blood.2022015970 35609283 10.1182/blood.2022015970 PMC9412009

[CR10] Canobbio I (2019) Blood platelets: Circulating mirrors of neurons? Res Pract Thromb Haemost 3:564–565. 10.1002/rth2.12254 31624775 10.1002/rth2.12254 PMC6781913

[CR11] Cipriano GL, Schepici G, Mazzon E, Anchesi I (2024) Multiple Sclerosis: roles of mirna, lcnrna, and circrna and their implications in cellular pathways. Int J Mol Sci 25:2255. 10.3390/ijms25042255 38396932 10.3390/ijms25042255 PMC10889752

[CR12] Comi G, Bar-Or A, Lassmann H, Uccelli A, Hartung HP, Montalban X et al (2021) Expert panel of the 27th annual meeting of the European Charcot foundation. Role of B cells in multiple sclerosis and related disorders. Ann Neurol 89:13–23. 10.1002/ana.2592 33091175 10.1002/ana.25927 PMC8007167

[CR13] Constantinescu CS, Farooqi N, O’Brien K, Gran B (2011) Experimental autoimmune encephalomyelitis (EAE) as a model for multiple sclerosis (MS). Br J Pharmacol 164:1079–1106. 10.1111/j.1476-5381.2011.01302.x 21371012 10.1111/j.1476-5381.2011.01302.x PMC3229753

[CR14] Davalos D, Mahajan KR, Trapp BD (2019) Brain fibrinogen deposition plays a key role in MS pathophysiology - Yes. Mult Scler 25:1434–1435. 10.1177/1352458519852723 31315512 10.1177/1352458519852723 PMC6750992

[CR15] Dedoni S, Scherma M, Camoglio C, Siddi C, Dazzi L, Puliga R et al (2023) An overall view of the most common experimental models for multiple sclerosis. Neurobiol Dis 184:106230. 10.1016/j.nbd.2023.106230 37453561 10.1016/j.nbd.2023.106230

[CR16] Du C, Liu C, Kang J, Zhao G, Ye Z, Huang S, Li Z, Wu Z, Pei G (2009) MicroRNA miR-326 regulates TH-17 differentiation and is associated with the pathogenesis of multiple sclerosis. Nature Immunol 10(12):1252–9. 10.1038/ni.1798 19838199 10.1038/ni.1798

[CR17] Engelhardt B, Ransohoff RM (2012) Capture, crawl, cross: the T cell code to breach the blood-brain barriers. Trends Immunol 33:579–589. 10.1016/j.it.2012.07.004 22926201 10.1016/j.it.2012.07.004

[CR18] Filippi M, Preziosa P, Barkhof F, Ciccarelli O, Cossarizza A, De Stefano N, Gasperini C, Geraldes R, Granziera C, Haider L, Lassmann H (2024) The ageing central nervous system in multiple sclerosis: the imaging perspective. Brain 147(11):3665–80. 10.1093/brain/awae251 39045667 10.1093/brain/awae251 PMC11531849

[CR19] Forbes JD, Van Domselaar G, Bernstein CN (2016) The gut microbiota in immune-mediated inflammatory diseases. Front Microbiol 7:1081. 10.3389/fmicb.2016.01081 27462309 10.3389/fmicb.2016.01081 PMC4939298

[CR20] Glatigny S, Bettelli E (2018) Experimental autoimmune encephalomyelitis (EAE) as animal models of multiple sclerosis (MS). Cold Spring Harb Perspect Med 8:a028977. 10.1101/cshperspect.a028977 29311122 10.1101/cshperspect.a028977 PMC6211376

[CR21] Gold R, Linington C, Lassmann H (2006) Understanding pathogenesis and therapy of multiple sclerosis via animal models: 70 years of merits and culprits in experimental autoimmune encephalomyelitis research. Brain 129:1953–1971 16632554 10.1093/brain/awl075

[CR22] Groppa S, Gonzalez-Escamilla G, Eshaghi A, Meuth SG, Ciccarelli O (2021) Linking immune-mediated damage to neurodegeneration in multiple sclerosis: could network-based MRI help?. Brain communications 3(4):fcab237. 10.1093/braincomms/fcab237 10.1093/braincomms/fcab237 PMC8557667 34729480

[CR23] Gros A, Ollivier V, Ho-Tin-Noe B (2015) Platelets in inflammation: regulation of leukocyte activities and vascular repair. Front Immunol 5:678. 10.3389/fimmun.2014.00678 25610439 10.3389/fimmu.2014.00678 PMC4285099

[CR24] Gul S, Smith AD, Thompson RHS, Wright HP, Zilkha KJ (1970) Fatty acid composition of phospholipids from platelets and erythrocytes in multiple sclerosis. J Neurol Neurosurg Psychiat 33:506–510 5505678 10.1136/jnnp.33.4.506 PMC493509

[CR25] Hall SM (1972) The effect of injections of lysophosphatidyl choline into white matter of the adult mouse spinal cord. J Cell Sci 10:535–542 5018033 10.1242/jcs.10.2.535

[CR26] Hamilton AM, Forkert ND, Yang R, Wu Y, Rogers JA, Yong VW, Dunn JF (2019) Central nervous system targeted autoimmunity causes regional atrophy: a 9.4 T MRI study of the EAE mouse model of Multiple Sclerosis. Scientific Rep 9(1):8488. 10.1038/s41598-019-44682-6 10.1038/s41598-019-44682-6 PMC6560061 31186441

[CR27] Han MH, Hwang SI, Roy DB, Lundgren DH, Price JV, Ousman SS et al (2008) Proteomic analysis of active multiple sclerosis lesions reveals therapeutic targets. Nature 451:1076–1083. 10.1038/nature06559 18278032 10.1038/nature06559

[CR28] Heng AHS, Han CW, Abbott C, McColl SR, Comerford I (2022) Chemokine-Driven Migration of Pro-Inflammatory CD4+ T Cells in CNS Autoimmune Disease. Front Immunol 13:817473. 10.3389/fimmu.2022.817473 35250997 10.3389/fimmu.2022.817473 PMC8889115

[CR29] Hoffmann N, Lachnit N, Streppel M, Witter B, Neiss WF, Gutinas-Lichius O, Angelov DN (2002) Increased expression of ICAM-1, VCAM-1, MCP-1, and MIP-1α by spinal perivascular macrophages during experimental allergic encephalomyelitis in rats. BMC Immunol 3:11. 10.1186/1471-2172-3-11 12196270 10.1186/1471-2172-3-11 PMC126207

[CR30] Howell OW, Schulz-Trieglaff EK, Carassiti D, Gentleman SM, Nicholas R, Roncaroli F et al (2014) Extensive grey matter pathology in the cerebellum in multiple sclerosis is linked to inflammation in the subarachnoid space. Neuropath App Neurobiol 41:798–813. 10.1111/nan.12199 10.1111/nan.12199 25421634

[CR31] Joseph R, Tsering C, Grunfeld S, Welch KM (1992a) Further studies on platelet-mediated neurotoxicity. Brain Res 577:268–275. 10.1016/0006-8993(92)90283-f 1376633 10.1016/0006-8993(92)90283-f

[CR32] Joseph R, Tsering C, Welch KM (1992b) Study of platelet-mediated neurotoxicity in rat brain. Stroke 23:394–398. 10.1161/01.str.23.3.394 1542902 10.1161/01.str.23.3.394

[CR33] Kap YS, Jagessar SA, Dunham J, At’Hart B (2016) The common marmoset as an indispensable animal model for immunotherapy development in multiple sclerosis. Drug discovery today. 21(8):1200–5. 10.1016/j.drudis.2016.03.014 27060373 10.1016/j.drudis.2016.03.014

[CR34] Kapur R, Zufferey A, Boilard E, Semple JW (2015) Nouvelle cuisine: platelets served with inflammation. J Immunol 194:5579–5587. 10.4049/jimmunol.1500259 26048965 10.4049/jimmunol.1500259

[CR35] Karpus WJ (2020) Cytokines and chemokines in the pathogenesis of experimental autoimmune encephalomyelitis. J Immunol 204:316–326. 10.4049/jimmunol.1900914 31907274 10.4049/jimmunol.1900914

[CR36] Kaushik DK, Bhattacharya A, Lozinski BM, Yong VW (2021) Pericytes as mediators of infiltration of macrophage in multiple sclerosis. J Neuroinflam 18:301. 10.1186/s12974-021-02358-x 10.1186/s12974-021-02358-x PMC8705458 34952601

[CR37] Kim Y, Park J, Choi YK (2019) The role of astrocytes in the central nervous system focused on BK channel and heme oxygenase metabolites: A review. Antioxidants 8:121. 10.3390/antiox8050121 31060341 10.3390/antiox8050121 PMC6562853

[CR38] Klineova S, Lublin F (2018) Clinical course of multiple sclerosis. Cold Spring Harb Perspect Med 8:a028928. 10.1101/cshperspect.a028928 29358317 10.1101/cshperspect.a028928 PMC6120692

[CR39] Kniewallner KM, Bessa de Sousa DM, Unger MS, Mrowetz H, Aigner L (2020) Platelets in amyloidogenic mice are activated and invade the brain. Front Neurosci 14:129. 10.3389/fnins.2020.00129 32194368 10.3389/fnins.2020.00129 PMC7063083

[CR40] Kocovski P, Jiang X, D’Souza CS, Li Z, Dang PT, Wang X et al (2019) Platelet depletion is effective in ameliorating anxiety-like behavior and reducing the pro-inflammatory environment in the hippocampus in murine experimental autoimmune encephalomyelitis. J Clin Med 8:162–177. 10.3390/jcm8020162 30717130 10.3390/jcm8020162 PMC6406682

[CR41] Kocovski P, Tabassum-Sheikh N, Marinis S, Dang PT, Hale MW, Orian JM (2021) Immunomodulation eliminates inflammation in the hippocampus in experimental autoimmune encephalomyelitis, but does not ameliorate anxiety-like behavior. Front Immunol 12:639650. 10.3389/fimmu.2021.639650 34177891 10.3389/fimmu.2021.639650 PMC8222726

[CR42] Kornfeld SF, Cummings SE, Fathi S, Bonin SR, Kothary R (2021) MiRNA-145-5p prevents differentiation of oligodendrocyte progenitor cells by regulating expression of myelin gene regulatory factor. J Cell Physiol 236:997–1012. 10.1002/jcp.29910 32602617 10.1002/jcp.29910

[CR43] Koudriavtseva T, Stefanile A, Fiorelli M, Lapucci C, Lorenzano S, Zannino S et al (2020) Coagulation/complement activation and cerebral hypoperfusion in relapsing-remitting multiple sclerosis. Front Immunol 11:548604. 10.3389/fimmu.2020.548604 33193314 10.3389/fimmu.2020.548604 PMC7655134

[CR44] Koupenova M, Livada AC, Morrell CN (2022) Platelet and megakaryocyte roles in innate and adaptive immunity. Circ Res 130:288–238. 10.1161/CIRCRESAHA.121.319821 35050690 10.1161/CIRCRESAHA.121.319821 PMC8852355

[CR45] Langer HF, Choi EY, Zhou H, Schleicher R, Chung KJ, Tang Z et al (2012) Platelets contribute to the pathogenesis of experiemntal autoimmune encephalomyelitis. Circ Res 110:1202–1210. 10.1161/CIRCRESAHA.111.256370 22456181 10.1161/CIRCRESAHA.111.256370 PMC3382058

[CR46] Lassmann H (2010) Axonal and neuronal pathology in multiple sclerosis: what have we learnt from animal models? Exp Neurol 225:2–8. 10.1016/j.expneurol.2009.10.009 19840788 10.1016/j.expneurol.2009.10.009

[CR47] Lassmann H (2019) Pathogenic mechanisms associated with different clinical courses of multiple sclerosis. Front Immunol 9:3116. 10.3389/fimmu.2018.03116 30687321 10.3389/fimmu.2018.03116 PMC6335289

[CR48] Lassmann H, van Horssen J, Mahad D (2012) Progressive multiple sclerosis: pathology and pathogenesis. Nat Rev Neurol 8:647–656. 10.1038/nrneurol.2012.168 23007702 10.1038/nrneurol.2012.168

[CR49] Leiter O, Walker T (2020) Platelets in neurodegenerative conditions-Friend or foe? Front Immunol 11:747. 10.3389/fimmun.2020.00747 32431701 10.3389/fimmu.2020.00747 PMC7214916

[CR50] Liu Q, Gao Q, Zhang Y, Li Z, Mei X (2017) MicroRNA-590 promotes pathogenic Th17 cell differentiation through targeting Tob1 and is associated with multiple sclerosis. Biochem Biophys Res Commun 493:901–908. 10.1016/j.bbrc.2017.09.123 28947212 10.1016/j.bbrc.2017.09.123

[CR51] Lock C, Hermans G, Pedotti R, Brendoaln A, Schadt E, Garren H et al (2002) Gene-microarray analysis of multiple sclerosis lesions yields new targets validated in autoimmune encephalomyelitis. Nat Med 8:500–508. 10.1038/nm0502-500 11984595 10.1038/nm0502-500

[CR52] Lucchinetti C, Bruck W, Parisi J, Scheithauer B, Rodriguez M, Lassmann H (2000) Heterogeneity of multiple sclerosis lesions: implications for the pathogenesis of demyelination. Ann Neurol 47:707–717. 10.1002/1531-8249(200006)47:6%3c707::aid-ana3%3e3.0.co;2-q 10852536 10.1002/1531-8249(200006)47:6<707::aid-ana3>3.0.co;2-q

[CR53] Mapunda JA, Tibar H, Wand R, Engelhardt B (2022) How does the immune system enter the brain? Front Immunol 13:805657. 10.3389/fimmu.2022.805657 35273596 10.3389/fimmu.2022.805657 PMC8902072

[CR54] Marcos-Ramiro B, Oliva Nacarino P, Serrano-Pertierra E, Blanco-Gelaz MÁ, Weksler BB, Romero IA, Couraud PO, Tuñón A, López-Larrea C, Millán J, Cernuda-Morollón E. Microparticles in multiple sclerosis and clinically isolated syndrome: effect on endothelial barrier function. BMC neuroscience. 2014;15:1-4. bmcneurosci.biomedcentral.com/articles/10.1186/1471-2202-15-110 10.1186/1471-2202-15-110 PMC4261570 25242463

[CR55] McRedmond JP, Park SD, Reilly DF, Coppinger JA, Maguire PB, Shields DC, Fitzgerald DJ (2004) Integration of proteomics and genomics in platelets: A profile of platelet proteins and platelet-specific genes. Mol Cell Proteomics 3:133–144. 10.1074/mcp.M300063-MCP200 14645502 10.1074/mcp.M300063-MCP200

[CR56] Miller DJ, Rodriguez M (1995) Spontaneous and induced remyelination in multiple sclerosis and the Theiler’s virus model of central nervous system demyelination. Microsc Res Tech 32:230–245. 10.1002/jemt.1070320306 8527857 10.1002/jemt.1070320306

[CR57] Monaghan KL, Zheng W, Hu G, Wan ECK (2019) Monocytes and monocyte-derived antigen-presenting cells have distinct gene signatures in experimental model of multiple sclerosis. Front Immunol 26(10):2779. 10.3389/fimmu.2019.02779 10.3389/fimmu.2019.02779 PMC6889845 31849962

[CR58] Morel A, Bijak M, Miller E, Rywaniak J, Miller S, Saluk J (2015) Relationship between the increased haemostatic properties of blood platelets and oxidative stress level in multiple sclerosis patients with the secondary progressive stage. Oxidative Med Cellul Longev 2015(1):240918. 10.1155/2015/240918 10.1155/2015/240918 PMC4429191 26064417

[CR59] Nagalla S, Shaw C, Kong X, Kondkar AA, Edelstein LC, Ma L et al (2011) Platelet microRNA-mRNA coexpression profiles correlate with platelet reactivity. Blood 117:5189–5197. 10.1182/blood-2010-09-299719 21415270 10.1182/blood-2010-09-299719 PMC3109541

[CR60] Nathanson MJ, Savitski P (1952) Platelet adhesive index studies in multiple sclerosis and other neurologic disorders. Bull NY Acad Med 28:462–468 PMC1877246 14935498

[CR61] Navarro S, Talucci I, Göb V, Hartmann S, Beck S, Orth V, Stoll G, Maric HM, Stegner D, Nieswandt B (2024) The humanized platelet glycoprotein VI Fab inhibitor EMA601 protects from arterial thrombosis and ischaemic stroke in mice. Eur Heart J 45(43):4582–97. 10.1093/eurheartj/ehae482 39150906 10.1093/eurheartj/ehae482 PMC11560278

[CR62] Nazir FH, Wiberg A, Müller M, Mangsbo S, Burman J. Antibodies from serum and CSF of multiple sclerosis patients bind to oligodendroglial and neuronal cell-lines. Brain Commun 2023;5(3):fcad164. 10.1093/braincomms/fcad164 10.1093/braincomms/fcad164 PMC10233900 37274830

[CR63] Neu CT, Gutschner T, Haemmerle M (2020) Post-transcriptional expression control in platelet biogenesis and function. Int J Mol Sci 21:7614. 10.3390/ijms21207614 33076269 10.3390/ijms21207614 PMC7589263

[CR64] Nicolai L, Pekayvaz K, Massberg S (2024) Platelets: Orchestrators of immunity in host defense and beyond. Immunity 57:957–972. 10.1016/j.immuni.2024.04.008 38749398 10.1016/j.immuni.2024.04.008

[CR65] O’Neill EE, Brock CJ, von Kriegsheim AF, Pearce AC, Dwek RA, Watson SP, Hebestreit HF (2000) Towards complete analysis of the platelet proteome. Proteomics 2:288–305. 10.1002/1615-9861(200203)2:3%3c288::aid-prot288%3e3.0.co;2-0 10.1002/1615-9861(200203)2:3<288::aid-prot288>3.0.co;2-0 11921445

[CR66] Ochoa-Repáraz J, Mielcarz DW, Haque-Begum S, Kasper LH (2010) Induction of a regulatory B cell population in experimental allergic encephalomyelitis by alteration of the gut commensal microflora. Gut Microbes 1:103–108. 10.4161/gmic.1.2.11515 21326918 10.4161/gmic.1.2.11515 PMC3023588

[CR67] Orian JM, Maxwell DL (2023) Cerebellar pathology in multiple sclerosis and experimental autoimmune encephalomyelitis: current status and future directions. J CNS Dis 15:1–15. 10.1177/11795735231211508 10.1177/11795735231211508 PMC10629308 37942276

[CR68] Orian JM, D’Souza CS, Kocovski P, Krippner G, Hale MW, Wang X, Peter K (2021) Platelets in multiple sclerosis: Early and central mediators of inflammation and neurodegeneration and attractive targets for molecular imaging and site-directed therapy. Front Immunol 19(12):620963. 10.3389/fimmu.2021.620963 10.3389/fimmu.2021.620963 PMC7933211 33679764

[CR69] Orian JM, Maxwell DL, Lim VJ (2023) Active Induction of a Multiple Sclerosis-Like Disease in Common Laboratory Mouse Strains. InNeurobiology: Methods Protoc 2746:179-200). New York, NY: Springer US. 10.1007/978-1-0716-3585-8_15 10.1007/978-1-0716-3585-8_15 38070090

[CR70] Papiri G, D’Andreamatteo G, Cacchiò G, Alia S, Silvestrini M, Paci C et al (2023) Multiple Sclerosis: Iiflammatory and neuroglial aspects. Curr Issues Mol Biol 45:1443–1470. 10.3390/cimb45020094 36826039 10.3390/cimb45020094 PMC9954863

[CR71] Parnell GP, Booth DR (2017) The multiple sclerosis (MS) genetic risk factors indicate both acquired and innate immune cell subsets contribute to MS pathogenesis and identify novel therapeutic opportunities. Front Immunol 8:425. 10.3389/fimmu.2017.00425 28458668 10.3389/fimmu.2017.00425 PMC5394466

[CR72] Petersen MA, Ryu J, Akassologlu K (2018) Fibrinogen in neurological diseases: mechanisms, imaging and therapeutics. Nat Rev Neurosci 19:283–330. 10.1038/nrn.2018.13 29618808 10.1038/nrn.2018.13 PMC6743980

[CR73] Pham H, Ng SW, Klopstein A, Ramp AA, Ayers MM, Orian JM (2008) The astrocytic response in early experimental autoimmune encephalomyelitis occurs across both grey and white matter compartments. J Neuroimmunol 208:30–39. 10.1016/j.jneuroim.2008.12.010 10.1016/j.jneuroim.2008.12.010 19195719

[CR74] Philp AR, Reyes CR, Mansilla J, Sharma A, Zhao C, Valenzuela-Krugmann C, Rawji KS, Martinez GA, Dimas P, Hinrichsen B, Ulloa-Leal C (2024) Circulating platelets modulate oligodendrocyte progenitor cell differentiation during remyelination. Elif ;12:RP91757. 10.7554/eLife.91757. 10.7554/eLife.91757 PMC11335344 39163103

[CR75] Pike SC, Welsh N, Linzey M, Gilli F (2022) Theiler’s virus-induced demyelinating disease as an infectious model of progressive multiple sclerosis. Front Mol Neurosci 15:1019799. 10.3389/fnmol.2022.1019799 36311024 10.3389/fnmol.2022.1019799 PMC9606571

[CR76] Plantone D, Inglese M, Salvetti M, Koudriavtseva T (2019) A perspective of coagulation dysfunction in multiple sclerosis and in experimental allergic encephalomyelitis. Front Neurol 9:1175. 10.3389/fneur.2018.01175 30692962 10.3389/fneur.2018.01175 PMC6340371

[CR77] Polfliet MMJ, van de Veerdonk F, Dopp EdA, van Kesteren-Hendrikx EML, van Rooijen N, Dijkstra CD, van den Berg TK (2002) The role of perivascular and meningeal macrophages in experimental allergic encephalomyelitis. J Neuroimmunol 122:1–8. 10.1016/s0165-5728(01)00445-3 11777538 10.1016/s0165-5728(01)00445-3

[CR78] Power CA, Clemetson JM, Clemetson KJ, Wells TN (1995) Chemokine and chemokine receptor mRNA expression in human platelets. Cytokine 7:479–482. 10.1006/cyto.1995.0065 8580362 10.1006/cyto.1995.0065

[CR79] Procaccini C, De Rosa V, Pucino V, Formisano L, Matarese G (2015) Animal models of Multiple Sclerosis. Eur J Pharmacol 759:182–191. 10.1016/j.ejphar.2015.03.042 25823807 10.1016/j.ejphar.2015.03.042 PMC7094661

[CR80] Purdel C, Margină D, Adam-Dima I, Ungurianu A (2023) The beneficial effects of dietary interventions on gut microbiota-an up-to-date critical review and future perspectives. Nutrients 15:5005. 10.3390/nu15235005 38068863 10.3390/nu15235005 PMC10708505

[CR81] Putman TJ (1937) Evidence of vascular occlusion in multiple sclerosis and encephalomyelitis. Arch Neuro Psychiat 37:1298–1313

[CR82] Robinson AP, Harp CT, Noronha A, Miller SD (2014) The experimental autoimmune encephalomyelitis (EAE) model of MS: utility for understanding disease pathophysiology and treatment. Handb Clin Neurol 122:173–189. 10.1016/B978-0-444-52001-2.00008-X 24507518 10.1016/B978-0-444-52001-2.00008-X PMC3981554

[CR83] Rondina MT, Garraud O (2014) Emerging evidence for platelets as immune and inflammatory effector cells. Front Immunol 5:653. 10.3389/fimmu.2014.00653 25566264 10.3389/fimmu.2014.00653 PMC4270189

[CR84] Ryu JK, Rafalski VA, Meyer-Franke A, Adams RA, Poda SB, Rios Coronado PE et al (2018) Fibrin-targeting immunotherapy protects against neuroinflammation and neurodegeneration. Nat Immunol 19:1212–1223. 10.1038/s41590-018-0232-x 30323343 10.1038/s41590-018-0232-x PMC6317891

[CR85] Saenz-Cuesta M, Osorio-Querejeta I, Otaegui D (2014) Extracellular vesicles in multiple sclerosis: what are they telling us? Front Cell Neurosci 8:100. 10.3389/fncel.2014.00100 24734004 10.3389/fncel.2014.00100 PMC3975116

[CR86] Saluk-Bijak J, Dziedzic A, Bijak M (2019) Pro-Thrombotic activity of blood platelets in multiple sclerosis. Cells 8:110. 10.3390/cells8020110 30717273 10.3390/cells8020110 PMC6406904

[CR87] Saxena A, Bauer J, Scheikl T, Zappulla J, Audebert M, Desbois S (2008) Cutting edge: Multiple sclerosis-like lesions induced by effector CD8 T cells recognizing a sequestered antigen on oligodendrocytes. J Immunol 181:1617–1621. 10.4049/jimmunol.181.3.1617 18641296 10.4049/jimmunol.181.3.1617

[CR88] Seifert HA, Benedek G, Nguyen H, Gerstner G, Zhang Y, Kent G (2018) Antibiotics protect against EAE by increasing regulatory and anti-inflammatory cells. Metab Brain Dis 33:1599–1607. 10.1007/s11011-018-0266-7 29916184 10.1007/s11011-018-0266-7 PMC6298859

[CR89] Smyth SS, McEver RP, Weyrich AS, Morrell CN, Hoffman MR, Arepally GM et al (2009) Platelet Colloquium Participants. Platelet functions beyond hemostasis. J Thromb Haemost 7:1759–1766. 10.1111/j.1538-7836.2009.03586.x 19691483 10.1111/j.1538-7836.2009.03586.x

[CR90] Sonia D’Souza CS, Li Z, Maxwell DL, Trusler O, Murphy M, Crewther S, Peter K, Orian JM (2018) Platelets drive inflammation and target gray matter and the retina in autoimmune-mediated encephalomyelitis. J Neuropathol Exp Neurol 7:567–576. 10.1093/jnen/nly032 10.1093/jnen/nly032 29757405

[CR91] Sotnikov I, Veremeyko T, Starossom SC , Barteneva N , Weiner HL, Ponomarev ED (2013) Platelets recognize brain-specific glycolipid structures, respond to neurovascular damage and promote neuroinflammation. PLoS One e58979. 10.1371/journal.pone.0058979. 10.1371/journal.pone.0058979 PMC3608633 23555611

[CR92] Starossom SC, Veremeyko T, Yung AW, Dukhinova M, Au C, Lau AY, et al. (2015) Platelets play differential role during the initiation and progression of autoimmune neuroinflammation. Circ Res 117:779–92. 10.1161/CIRCRESAHA.115.306847. 10.1161/CIRCRESAHA.115.306847 PMC4716010 26294656

[CR93] Sulimai N, Lominadze D (2021) Fibrinogen and/or fibrin as a cause of neuroinflammation. Online J Neurol Brain Disord 5:217 34327331 PMC8318361

[CR94] Sun D, Whitaker JN, Huang Z, Liu D, Coleclough C, Wekerle H, Raine CS (2001) Myelin antigen-specific CD8+ T cells are encephalitogenic and produce severe disease in C57BL/6 mice. J Immunol 166:7579–7587. 10.4049/jimmunol.166.12.7579 11390514 10.4049/jimmunol.166.12.7579

[CR95] Swan D, Loughranb N, Makrisc M, Thachilb J (2020) Management of bleeding and procedures in patients on antiplatelet therapy. Blood Rev 39:100619. 10.1016/j.blre.2019.100619 31648803 10.1016/j.blre.2019.100619

[CR96] ‘t hart BA, van Meurs M, Brok HP et al (2000) A new primate model for multiple sclerosis in the common marmoset. Immunol Today 21:290–297. 10.1016/s0167-5699(00)01627-3 10825741 10.1016/s0167-5699(00)01627-3

[CR97] Thompson JW, Hu R, Huffaker TB, Ramstead AG, Ekiz HA, Bauer KM et al (2023) MicroRNA-155 plays selective cell-intrinsic roles in brain-infiltrating immune cell populations during neuroinflammation. J Immunol 210:926–934. 10.4049/jimmunol.2200478 36883849 10.4049/jimmunol.2200478 PMC10305808

[CR98] Török O, Schreiner B, Schaffenrath J, Tsai HC, Maheshwari U, Stifter SA et al (2021) Pericytes regulate vascular immune homeostasis in the CNS. Proc Natl Acad Sci U S A 118:e2016587118. 10.1073/pnas.2016587118 33653955 10.1073/pnas.2016587118 PMC7958247

[CR99] Vistbakka J, Sumelahti M-L, Lehtimaki T, Hagman S (2022) Temporal variability of serum miR-191, miR-223, miR-128, and miR-24 in multiple sclerosis: A 4-year follow-up study. J Neurol Sci 4421:120395. 10.1016/j.jns.2022.120395 10.1016/j.jns.2022.120395 36084364

[CR100] Voskuhl RR, MacKenzie-Graham A (2022) Chronic experimental autoimmune encephalomyelitis is an excellent model to study neuroaxonal degeneration in multiple sclerosis. Front Mol Neurosci 15:1024058. 10.3389/fnmol.2022.1024058 36340686 10.3389/fnmol.2022.1024058 PMC9629273

[CR101] Wachowicz B, Morel A, Miller E, Saluk J (2016) The physiology of blood platelets and changes of their biological activities in multiple sclerosis. Acta Neurobiologiae Experimentalis 76(4):269-81. 10.21307/ane-2017-026 10.21307/ane-2017-026 28094818

[CR102] Wang S, Chen H, Wen X, Mu J, Sun M, Song X et al (2021) The Efficacy of Fecal Microbiota Transplantation in Experimental Autoimmune Encephalomyelitis: Transcriptome and Gut Microbiota Profiling. J Immunol 2021:1–12. 10.1155/2021/4400428 10.1155/2021/4400428 PMC8687821 34938813

[CR103] Wassmer SC, Humpel C, Orian JM (2021) Platelets as Players in Neuropathologies: Novel Diagnostic and Therapeutic Targets. Front Immunol 12:772352. 10.3389/fimmu.2021.772352 34659273 10.3389/fimmu.2021.772352 PMC8513524

[CR104] Wen T, Zhang Z (2023) Cellular mechanisms of fibrin(ogen): insight from neurodegenerative diseases. Front Neurosci 17:1197094. 10.3389/fnins.2023.1197094 37529232 10.3389/fnins.2023.1197094 PMC10390316

[CR105] Weyrich AS, Zimmermann GA (2004) Platelets: signaling cells of the immune continuum. Trends Immunol 25:489–495. 10.1016/j.it.2004.07.003 15324742 10.1016/j.it.2004.07.003

[CR106] Woo MS, Engler JB, Friese MA (2024) The neuropathobiology of multiple sclerosis. Nat Rev Neurosci 25:493–513. 10.1038/s41583-024-00823-z 38789516 10.1038/s41583-024-00823-z

[CR107] Wright HP, Thompson RHS, Zilka KJ (1965) Platelet adhesiveness in multiple scerosis. Lancet 2:1109–1110 4158810 10.1016/s0140-6736(65)90069-3

[CR108] Wu H, Deng R, Chen X, Wong WC, Chen H, Gao L, Nie Y, Wu W, Shen J (2016) Caveolin-1 is critical for lymphocyte trafficking into central nervous system during experimental autoimmune encephalomyelitis. J Neurosci 36(19):5193–9. 10.1523/JNEUROSCI.3734-15.2016 27170118 10.1523/JNEUROSCI.3734-15.2016 PMC6601805

[CR109] Xu J, Ganguly A, Zhao J, Ivey M, Lopez R, Osterholzer JJ, Cho CS, Olszewski MA (2021) CCR2 signaling promotes brain infiltration of inflammatory monocytes and contributes to neuropathology during cryptococcal meningoencephalitis. MBio 12(4):10–128. 10.1128/mBio.01076-21 10.1128/mBio.01076-21 PMC8406332 34311579

[CR110] Zierfuss B, Larochelle C, Prat A (2024) Blood-brain barrier dysfunction in multiple sclerosis: causes, consequences and potential effects on therapies. Lancet Neurol 23:95–109 38101906 10.1016/S1474-4422(23)00377-0

[CR111] Ziliotto N, Bernrdi F, Jakimovski D, Zivadinov R (2019) Coagulation pathways in neurological diseases: Multiple sclerosis. Front Neurol 10:409. 10.3389/fneur.2019.00409 31068896 10.3389/fneur.2019.00409 PMC6491577

[CR112] Zirngibl M, Assinck P, Sizov A, Caprariello AV, Plemel JR (2022) Oligodendrocyte death and myelin loss in the cuprizone model: an updated overview of the intrinsic and extrinsic causes of cuprizone demyelination. Mol Neurodegener 17:34. 10.1186/s13024-022-00538-8 35526004 10.1186/s13024-022-00538-8 PMC9077942

